# An incidental finding of a large genomic deletion of BRCA1 on a molecular karyotype for a 5 year old child

**DOI:** 10.1186/1897-4287-10-S2-A73

**Published:** 2012-04-12

**Authors:** A Lewis, P James

**Affiliations:** 1 Peter MacCallum Familial Cancer Centre, Melbourne, Victoria, Australia

## Background

The use of microarray based molecular karyotyping for diagnostic testing is now common in clinical practice. A feature of this technology is an increased capacity to uncover genetic abnormalities unrelated to the original indication for the test. These incidental findings can involve the unexpected diagnosis of well recognised Mendelian genetic disorders through the rearrangement of a known disease genes. Large scale genomic rearrangements of *BRCA1* make a significant contribution to the molecular pathology of familial breast and ovarian cancer and are potentially detectable by the CGH or SNP microarrays in common use.

## Case report

We describe the case of a 5 year old boy seen at the Peter MacCallum Familial Cancer Centre. The index case and his twin brother originally presented to their pediatrician for investigation of a history of an autistic spectrum disorder and a molecular karyotype was ordered. This array revealed an unexpected finding of a deletion on chromosome 17q21 involving the *BRCA1* gene locus. Analysis showed that the copy number variant resulted in the deletion of exons 2-17 and would be classified as a pathogenic mutation. Following discussion with a clinical geneticist the result was reported to the family by the pediatrician who facilitated their referral them to a familial cancer centre for further discussion.

A detailed pedigree revealed no significant cancer history with only a single case of breast cancer in the wider family. The parents of the index described being surprised by the result but grateful for the information. Both parents elected to proceed with predictive testing to clarify the origin of the mutation and the potential cancer risks for them and their family in the future.

## Conclusion

The rapid expansion of genetic testing capacity through new technologies such as microarray testing or next-Generation-Sequencing are certain to be associated with increasing numbers of unexpected findings including familial cancer syndromes. Although an approach to this type of finding needs to be developed, the current case demonstrates that whilst unanticipated, this information can be welcomed by families.

